# Involvement of spinal NR2B-containing NMDA receptors in oxaliplatin-induced mechanical allodynia in rats

**DOI:** 10.1186/1744-8069-7-8

**Published:** 2011-01-20

**Authors:** Yuki Mihara, Nobuaki Egashira, Hikaru Sada, Takehiro Kawashiri, Soichiro Ushio, Takahisa Yano, Hiroaki Ikesue, Ryozo Oishi

**Affiliations:** 1 Department of Pharmacy, Kyushu University Hospital, 3-1-1 Maidashi, Higashi-ku, Fukuoka 812-8582, Japan

## Abstract

**Background:**

Oxaliplatin is a platinum-based chemotherapy drug characterized by the development of acute and chronic peripheral neuropathies. The chronic neuropathy is a dose-limiting toxicity. We previously reported that repeated administration of oxaliplatin induced cold hyperalgesia in the early phase and mechanical allodynia in the late phase in rats. In the present study, we investigated the involvement of NR2B-containing N-methyl-D-aspartate (NMDA) receptors in oxaliplatin-induced mechanical allodynia in rats.

**Results:**

Repeated administration of oxaliplatin (4 mg/kg, i.p., twice a week) caused mechanical allodynia in the fourth week, which was reversed by intrathecal injection of MK-801 (10 nmol) and memantine (1 μmol), NMDA receptor antagonists. Similarly, selective NR2B antagonists Ro25-6981 (300 nmol, i.t.) and ifenprodil (50 mg/kg, p.o.) significantly attenuated the oxaliplatin-induced pain behavior. In addition, the expression of NR2B protein and mRNA in the rat spinal cord was increased by oxaliplatin on Day 25 (late phase) but not on Day 5 (early phase). Moreover, we examined the involvement of nitric oxide synthase (NOS) as a downstream target of NMDA receptor. L-NAME, a non-selective NOS inhibitor, and 7-nitroindazole, a neuronal NOS (nNOS) inhibitor, significantly suppressed the oxaliplatin-induced pain behavior. The intensity of NADPH diaphorase staining, a histochemical marker for NOS, in the superficial layer of spinal dorsal horn was obviously increased by oxaliplatin, and this increased intensity was reversed by intrathecal injection of Ro25-6981.

**Conclusion:**

These results indicated that spinal NR2B-containing NMDA receptors are involved in the oxaliplatin-induced mechanical allodynia.

## Background

Glutamate is a major excitatory transmitter in the spinal cord and N-methyl-D-aspartate (NMDA) receptors are known to be involved in the painful neuropathy [1,2]. The NMDA receptor antagonist inhibits the pain hypersensitivity in chronic constriction injury (CCI) model. Moreover, the expression of spinal NR2B-containing NMDA receptors is increased with the pain hypersensitivity induced by CCI or chronic compression of the dorsal root ganglia (CCD) [3-6]. Selective NR2B antagonists inhibit mechanical allodynia without causing motor dysfunction in CCI, CCD and spinal nerve-ligated (SNL) neuropathic models [5-8]. In addition, the NR2B subunits are localized to the superficial dorsal horn of the spinal cord [7,9], suggesting a possible involvement in pain transmission. Thus, the NR2B-containing NMDA receptors may play an important role in the neuropathic pain.

Nitric oxide synthase (NOS) as a downstream target of NMDA receptor also contributes greatly to the incidence of neuropathic pain. In the rat CCI model of neuropathic pain, intrathecal injection of non-selective NOS inhibitor L-N^G^-nitro-arginine methyl ester (L-NAME) reverses the persistent thermal hyperalgesia [10]. Furthermore, a close correlation between neuronal NOS (nNOS) and neuropathic pain has been documented in CCI model [11].

Oxaliplatin, a third-generation platinum-based chemotherapy drug, has widely been used as a key drug in the treatment of colorectal cancer. However, oxaliplatin causes severe acute and chronic peripheral neuropathies. The acute neuropathy includes acral paresthesias and dysesthesia triggered or enhanced by exposure to cold, and it appears soon after administration [12]. After multiple cycles the patients develop the chronic neuropathy that is characterized by a sensory and motor dysfunction. This chronic neuropathy is a dose-limiting toxicity and a major clinical problem in oxaliplatin chemotherapy [13].

Recently, we reported that repeated administration of oxaliplatin induced cold hyperalgesia in the early phase and mechanical allodynia in the late phase in rats [14]. Oxaliplatin is metabolized to oxalate and dichloro(1,2-diaminocyclohexane)platinum [Pt(dach)Cl_2_] [15]. We demonstrated that oxalate and platinum metabolite are involved in the cold hyperalgesia and mechanical allodynia, respectively [14]. Oxalate alters voltage-gated Na^+ ^channels [16] and its effect may be involved in the cold hyperalgesia. On the other hand, the mechanical allodynia may be due to the peripheral nerve injury by platinum metabolite. However, whether the NR2B-containing NMDA receptors are involved still remains largely unclear. In the present study, we investigated the involvement of NR2B-containing NMDA receptors in the oxaliplatin-induced mechanical allodynia in rats.

## Results

### Effects of NMDA receptor antagonists on oxaliplatin-induced mechanical allodynia

**Figure 1:**
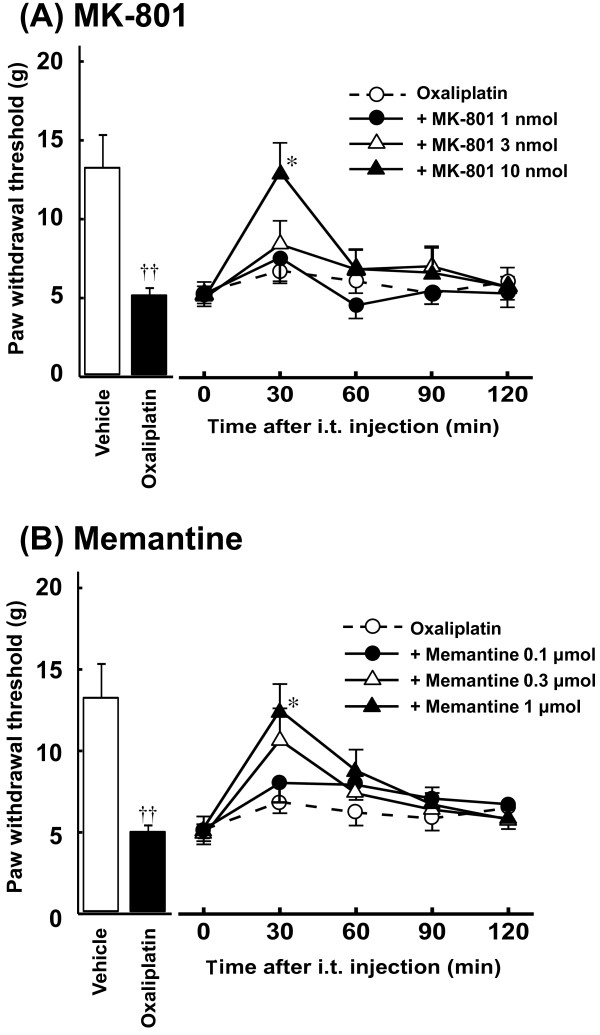
**Effects of MK-801 (A) and memantine (B) on oxaliplatin-induced mechanical allodynia in the von Frey test**. Oxaliplatin (4 mg/kg) was administered i.p. twice a week for 4 weeks (Days 1, 2, 8, 9, 15, 16, 22 and 23). We confirmed the incidence of mechanical allodynia on Day 24. We carried out the drug evaluation on the next day. MK-801 (1-10 nmol/body) and memantine (0.1-1 μmol/body) were administered intrathecally. The von Frey test was performed immediately before (0 min) and at 30, 60, 90 and 120 min after administration. Values are expressed as mean ± SEM of 6-10 animals. ^††^*P *< 0.01 compared with vehicle, *P < 0.05 compared with oxaliplatin alone.

Oxaliplatin (4 mg/kg, i.p., twice a week for 4 weeks) significantly reduced the paw withdrawal thresholds compared with the vehicle in the von Frey test on Day 24 (*P *< 0.01, Figure 1). Acute administration of a NMDA receptor antagonist MK-801 (10 nmol, i.t.) completely reversed the reduction of paw withdrawal threshold by oxaliplatin at 30 min after administration (*P *< 0.05, Figure 1A). Similarly, acute administration of another NMDA receptor antagonist memantine (1 μmol, i.t.) completely reversed the reduction of paw withdrawal threshold by oxaliplatin at 30 min after administration (*P *< 0.05, Figure 1B). These effects of MK-801 and memantine disappeared by 120 min after administration. In addition, MK-801 (10 nmol, i.t.) and memantine (1 μmol, i.t.) had no effect on the paw withdrawal thresholds in intact rats (data not shown).

### Effects of NR2B antagonists on oxaliplatin-induced mechanical allodynia

**Figure 2:**
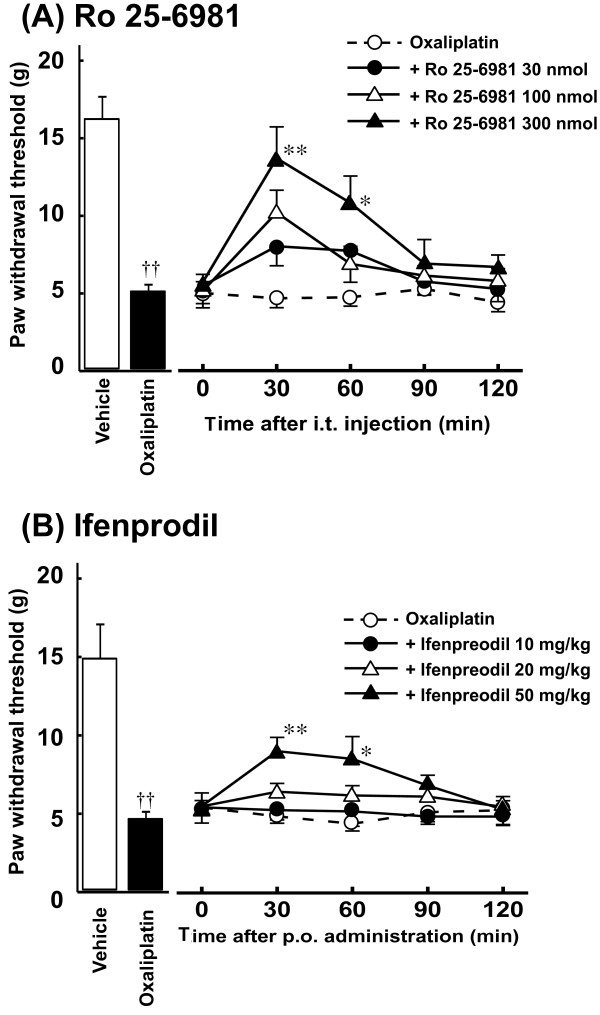
**Effects of Ro 25-6981 (A) and ifenprodil (B) on oxaliplatin-induced mechanical allodynia in the von Frey test**. Oxaliplatin (4 mg/kg) was administered i.p. twice a week for 4 weeks (Days 1, 2, 8, 9, 15, 16, 22 and 23). We confirmed the incidence of mechanical allodynia on Day 24. We carried out the drug evaluation on the next day. Ro 25-6981 (30-300 nmol/body) was administered intrathecally. Ifenprodil (10-50 mg/kg) was administered orally. The von Frey test was performed immediately before (0 min) and at 30, 60, 90 and 120 min after administration. Values are expressed as mean ± SEM of 6-7 animals. ^††^*P *< 0.01 compared with vehicle, *P < 0.05, **P < 0.01 compared with oxaliplatin alone.

Acute administration of a selective NR2B antagonist Ro 25-6981 (300 nmol, i.t.) significantly inhibited the reduction of paw withdrawal threshold by oxaliplatin at 30 and 60 min after administration (*P *< 0.01: 30 min, *P *< 0.05: 60 min, Figure 2A). Similarly, acute administration of another NR2B antagonist ifenprodil (50 mg/kg, p.o.) significantly inhibited the reduction of paw withdrawal threshold by oxaliplatin at 30 and 60 min after administration (*P *< 0.01: 30 min, *P *< 0.05: 60 min, Figure 2B). These effects of Ro 25-6981 and ifenprodil disappeared by 120 min after administration. In addition, Ro 25-6981 (300 nmol, i.t.) and ifenprodil (50 mg/kg, p.o.) had no effect on the paw withdrawal thresholds in intact rats (data not shown).

### Changes of NR2B protein and mRNA in the spinal cord in oxaliplatin-treated rats

**Figure 3:**
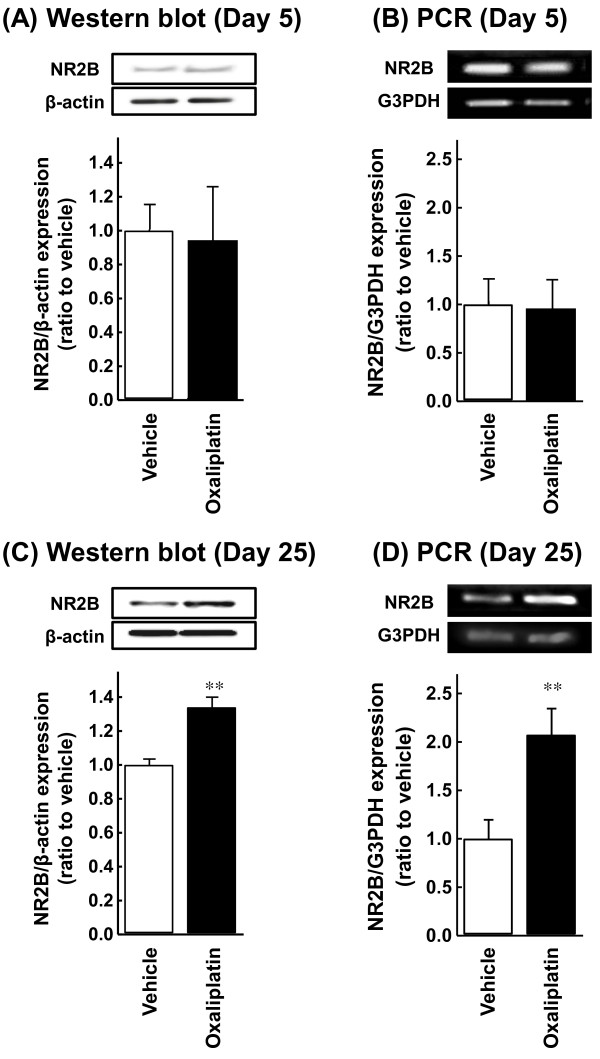
**Changes in the NR2B protein (A, C) and mRNA (B, D) levels in the spinal cord in oxaliplatin-treated rats**. Oxaliplatin (4 mg/kg) was administered i.p. twice a week for 1 (Days 1 and 2) or 4 weeks (Days 1, 2, 8, 9, 15, 16, 22 and 23). Western blot and PCR were performed to measure NR2B expression at the time points of Days 5 (A, B) and 25 (C, D) in the L4-6 spinal cord. Values are expressed as mean ± SEM of 6-9 animals. **P < 0.01 compared with vehicle.

NR2B expression was examined by Western blot and polymerase chain reaction (PCR) analysis on homogenates of the spinal cord from rats. The results of Western blot and PCR showed that NR2B protein and mRNA levels in the spinal cord of oxaliplatin-treated rats significantly increased compared with that of vehicle-treated rats on Day 25 (*P *< 0.01, Figures 3C, D). On the other hand, oxaliplatin caused no change in NR2B protein and mRNA levels in the spinal cord on Day 5 (Figures 3A, B).

### Effects of NOS inhibitors on oxaliplatin-induced mechanical allodynia

**Figure 4:**
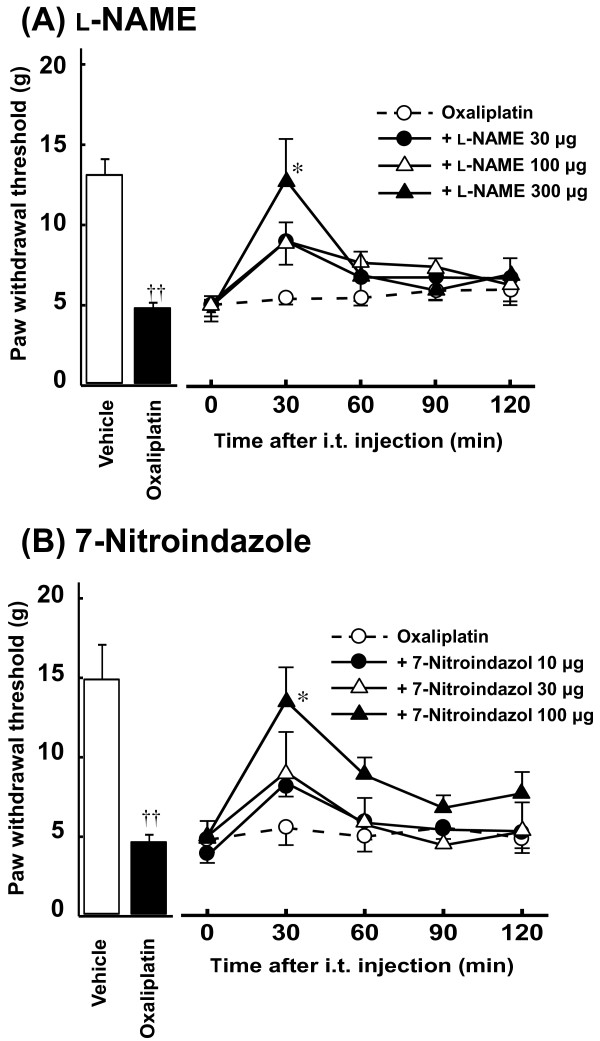
**Effects of L-NAME (A) and 7-nitroindazole (B) on oxaliplatin-induced mechanical allodynia in the von Frey test**. Oxaliplatin (4 mg/kg) was administered i.p. twice a week for 4 weeks (Days 1, 2, 8, 9, 15, 16, 22 and 23). We confirmed the incidence of mechanical allodynia on Day 24. We carried out the drug evaluation on the next day. L-NAME (30-300 μg/body) and 7-nitroindazole (10-100 μg/body) were administered intrathecally. The von Frey test was performed immediately before (0 min) and at 30, 60, 90 and 120 min after administration. Values are expressed as mean ± SEM of 6-10 animals. ^††^*P *< 0.01 compared with vehicle, *P < 0.05 compared with oxaliplatin alone.

Acute administration of a non-selective NOS inhibitor L-NAME (300 μg, i.t.) completely reversed the reduction of paw withdrawal threshold by oxaliplatin at 30 min after administration (*P *< 0.05, Figure 4A). Similarly, acute administration of an nNOS inhibitor 7-nitroindazole (100 μg, i.t.) significantly inhibited the reduction of paw withdrawal threshold by oxaliplatin at 30 min after administration (*P *< 0.05, Figure 4B). These effects of L-NAME and 7-nitroindazole disappeared by 120 min after administration. In addition, L-NAME (300 μg, i.t.) and 7-nitroindazol (100 μg, i.t.) had no effect on the paw withdrawal thresholds in intact rats (data not shown).

### Change of NOS activity in the spinal cord of oxaliplatin-treated rats

**Figure 5:**
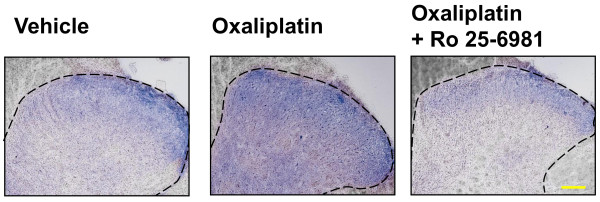
**Typical photomicrographs representing NOS histochemistry staining of neurons in the spinal cord**. Oxaliplatin (4 mg/kg) was administered i.p. twice a week for 4 weeks (Days 1, 2, 8, 9, 15, 16, 22 and 23). On Day 25, Ro 25-6981 (300 nmol/body) was administered intrathecally, and the L4-6 spinal cord was removed 30 min after administration. NADPH diaphorase staining was performed to confirm NOS activity. The blue staining shows NADPH diaphorase staining. Spinal slices (30 μm) were prepared from rats treated with vehicle (A), oxaliplatin alone (B), and oxaliplatin and Ro 25-6981 (C). Scale bar, 200 μm.

To evaluate change of NOS activity in oxaliplatin-induced mechanical allodynia, we carried out the NADPH diaphorase staining, a histochemical marker for NOS, in rat spinal cord sections. The results of NADPH diaphorase histochemistry revealed that the intensity of NADPH diaphorase staining (blue staining) obviously increased in the superficial layer of spinal dorsal horn in oxaliplatin-treated rats on Day 25 (Figure 5). Moreover, this increased intensity was reversed by intrathecal injection of Ro 25-6981 (300 nmol).

## Discussion

In this study, NMDA receptor antagonists completely reverse the oxaliplatin-induced mechanical allodynia when administered after the development of neuropathy. Similarly, selective NR2B antagonists significantly inhibited the oxaliplatin-induced mechanical allodynia. Moreover, the expression of NR2B protein and mRNA in the spinal cord increased in the oxaliplatin-treated rats on Day 25 (late phase) but not on Day 5 (early phase). Oxaliplatin (4 mg/kg, i.p., twice a week) induces cold hyperalgesia in the early phase and mechanical allodynia in the late phase [14]. These findings suggest that the up-regulation of spinal NR2B-containing NMDA receptors is involved in the incidence of mechanical allodynia by repeated administration of oxaliplatin.

To investigate whether spinal cord NOS as the downstream target of NMDA receptor contributes to the incidence of mechanical allodynia, we examined the effects of NOS inhibitors on the oxaliplatin-induced mechanical allodynia. Intrathecal injection of L-NAME, a non-selective NOS inhibitor, and 7-nitroindazole, a selective nNOS inhibitor, inhibited the pain behavior, suggesting that NOS especially nNOS is involved in the oxaliplatin-induced mechanical allodynia. This is further supported by the finding that the intensity of NADPH diaphorase staining in the rat spinal dorsal horn was increased by repeated administration of oxaliplatin, and that this increased intensity was reversed by intrathecal injection of Ro25-6981, which attenuated the oxaliplatin-induced pain behavior. Marked increase of nNOS expression in the dorsal root ganglia (DRG) and spinal cord contributes to spinal sensory processing in CCI model [11]. More recent experiments with selective NOS inhibitors and in NOS-deficient mice revealed the nNOS to be the most important NO-producing enzyme in the spinal cord during the development and maintenance of neuropathic pain in SNL model [17]. In mice with neuropathic pain by transection of spinal nerve, an increase in nNOS activity is visualized in the superficial dorsal horn by NADPH diaphorase histochemistry [18]. Taken together, these findings suggest that NOS especially nNOS contributes to the incidence of oxaliplatin-induced mechanical allodynia.

Interestingly, our results show that both oxaliplatin-induced pain behavior and increase of NOS activity are reversed by intrathecal injection of Ro25-6981, a selective NR2B antagonist. Phosphorylation of NMDA receptor NR2B subunits increases nNOS activity in the superficial dorsal horn of mice with neuropathic pain [18]. The activation of NMDA receptor also induces glutamate release through NOS activity [19]. Thus, the NMDA receptor and NOS comprise a local circuit that amplifies the signal of pain transmission. If sustained production of these factors by repeated administration of oxaliplatin is required for maintenance of mechanical allodynia, and if their treatment-induced increase is likely to cause persistence of pain, blockade of this circuit by the NR2B antagonist would likely reduce pain excitatory neurotransmission in the spinal cord. All of these findings indicate that NR2B antagonists have analgesic effects on the oxaliplatin-induced mechanical allodynia at the spinal level.

Non-competitive NMDA receptor antagonists are used as analgesics in clinical practice, although undesirable side effects limit their utility [20]. In contrast, the restricted distribution of NR2B receptor makes them promising candidates as targets of side effect-free analgesic drugs [21]. Indeed, ifenprodil, traxoprodil (CP-101606) and Ro25-6981 are effective in inflammatory and/or neuropathic pain models in animals at doses that are not accompanied by motor effects [8,22]. In addition, ifenprodil has been used as analgesic adjuvant in clinical settings. In this study, our results showed that NMDA receptor antagonists, selective NR2B antagonists and NOS inhibitors at the effective dose had no effect on pain behavior in intact rats. Therefore, the ameliorative effects of these drugs were not attributable to non-specific sedative effects or a deficit of motor function, suggesting that the reduced pain behavior reflects a therapeutic effect on oxaliplatin-induced mechanical allodynia. Novel strategies involving NR2B antagonists may be a useful alternative or adjunct therapy for oxaliplatin-induced peripheral neuropathy.

## Conclusion

Our results indicate that repeated administration of oxaliplatin induces NR2B and NOS up-regulation in the spinal cord. This up-regulation may contribute to the incidence of mechanical allodynia. Furthermore, NMDA receptor antagonists, selective NR2B antagonists and NOS inhibitors remarkably attenuated the oxaliplatin-induced pain behavior. In addition, the selective NR2B antagonist inhibited the increase of NOS activity in the spinal cord. These results suggest that activation of the NMDA-NOS pathway contributes to the incidence of mechanical allodynia induced by repeated administration of oxaliplatin.

## Methods

### Animals

Male Sprague-Dawley rats weighing 200-250 g (Kyudo Co., Saga, Japan) were used in the present study. Rats were housed in groups of four to five per cage, with lights on from 7:00 to 19:00 h. Animals had free access to food and water in their home cages. All experiments were approved by the Experimental Animal Care and Use Committee of Kyushu University according to the National Institutes of Health guidelines, and we followed International Association for the Study of Pain (IASP) Committee for Research and Ethical Issues guidelines for animal research [23].

### Production of neuropathy

Mechanical allodynia was induced according to the method described previously [24]. Oxaliplatin (Elplat^®^) was obtained from Yakult Co., Ltd. (Tokyo, Japan). Oxaliplatin was dissolved in 5% glucose solution. The vehicle-treated rats were injected with 5% glucose solution. Oxaliplatin (4 mg/kg) or vehicle (5% glucose) was injected i.p. in volumes of 1 mL/kg twice a week for 4 weeks.

### von Frey test

The mechanical allodynia was assessed by von Frey test. Each rat was placed in a clear plastic box (20 × 17 × 13 cm) with a wire mesh floor and allowed to habituate for 30 min prior to testing. von Frey filaments (The Touch Test Sensory Evaluator Set; Linton Instrumentation, Norfolk, UK) ranging 1-15 g bending force were applied to the midplantar skin of each hind paw with each application held for 6 s. The paw withdrawal threshold was determined by a modified up-down method [25].

### Pharmacological studies

We confirmed the incidence of mechanical allodynia on Day 24. We carried out the drug evaluation on the next day. The von Frey test was performed immediately before (0 min) and at 30, 60, 90, and 120 min after administration. (+)-MK-801 maleate (Wako Pure Chemical Industries, Ltd., Osaka, Japan), memantine hydrochloride (Alexis Biochemicals, San Diego, CA, USA) and L-NAME (Sigma-Aldrich, Missouri, USA) were dissolved in saline and administered i.t. Ro 25-6981 (Sigma-Aldrich) and 7-nitroindazole were dissolved in 100% dimethyl sulfoxide (DMSO) and administered i.t. Ifenprodil tartrate (Wako Pure Chemical Industries, Ltd.) was suspended in 5% gum arabic solution and administered orally (p.o.). The doses of these drugs were chosen based on previous reports [8,26-28]. Behavioral test was performed blindly with respect to drug administration.

### Western blotting

To investigate the functional changes in protein levels of NR2B, the L4-6 spinal cord was quickly removed on Days 5 and 25. The tissues were homogenized in a solubilization buffer containing 20 mM Tris-HCl (pH7.4, 2 mM EDTA, 0.5 mM EGTA, 10 mM NaF, 1 mM Na_3_VO_4_, 1 mM PMSF, 0.32 M Sucrose, 2 mg/ml aprotinine, 2 mg/ml leupeptin), and the homogenates were subjected to 6% SDS-PAGE, and proteins were transferred electrophoretically to PVDF membranes. The membranes were blocked in Tris-buffered saline Tween-20 (TBST) containing 5% BSA (Sigma-Aldrich) for an additional 1 h at room temperature with agitation. The membrane was incubated overnight at 4°C with rabbit polyclonal NR2B antibody (1:5000; Upstate Biotech, NY, USA) and then incubated for 1 h with anti-rabbit IgG horseradish peroxidase (1:5000; Jackson Immuno Research Laboratories, Inc., PA, USA). The immunoreactivity was detected using Enhanced Chemiluminescence (Perkin Elmer, Massachusetts, USA).

### Reverse transcriptase-polymerase chain reaction (RT-PCR)

To investigate the functional changes in mRNA levels of NR2B, the L4-6 spinal cord was quickly removed on Days 5 and 25. mRNA was isolated using PolyATtract^® ^System 1000 (Promega, Corp., Wisconsin, USA). cDNA was synthesized using PrimScript^® ^1st strand cDNA Synthesis Kit (TaKaRa Bio, Inc., Shiga, Japan). PCR was carried out with Gene Taq (Nippon Gene, Co., Ltd., Tokyo, Japan). The oligonucleotide primers for NR2B were designed based on the sequences described by Lau et al. [29]. The sequences of PCR primers were as follows: NR2B, 5'-TCC GTC TTT CTT ATG TGG ATA TGC-3' (sense), 5'-CCT CTA GGC GGA CAG ATT AAG G-3' (antisense); glyceraldehyde-3-phosphate dehydrogenase (G3PDH), 5'-YGC CTG CTT CAC CAC CTT-3' (sense), 5'-TGC MTC CTG CAC CAC CAA CT-3' (antisense) (Sigma-Aldrich). Reactions were run for 40 cycles with 95°C denaturing cycle (30 s), 63°C annealing cycle (1 min) and 72°C extension cycle (30 s) for NR2B or for 30 cycles with 94°C denaturing cycle (45 s), 53°C annealing cycle (45 s) and 72°C extension cycle (1.5 min) for G3PDH, respectively. The PCR products were subjected to electrophoresis on 2% agarose gel, and the DNA was visualized by staining with ethidium bromide under ultraviolet irradiation. Then, the intensities of PCR products were semi-quantified densitometrically by Alpha Imager 2200 (Cell Biosciences, Inc., California, USA).

### NADPH diaphorase histochemistry

Animals were anaesthetized with pentobarbital (50 mg/kg) and perfused through the left cardiac ventricle with 50 mL physiological saline followed by a fixative containing 4% paraformaldehyde in 0.1 M sodium phosphate (pH 7.4). The L4-6 spinal cord was removed and immersed in the fixative for 4 h and then cryoprotected overnight in 30% (w/v) sucrose in 0.1 M phosphate-buffered saline (pH 7.4). Transverse frozen sections (30 μm) were cut on a cryostat. These sections were thaw-mounted on slides and NOS activity was determined using NADPH diaphorase histochemistry as described by Mabuchi et al. [30]. The incubation was performed for 1 h at 37°C in a reaction mixture containing 0.5 mg/mL β-NADPH, 0.2 mg/mL nitroblue tetrazolium and 0.25% Triton X-100 in 0.1 M phosphate-buffered saline (pH 7.4).

### Statistical analyses

Values were expressed as mean ± SEM. Results were analyzed by Student's *t*-test or one-way analysis of variance (ANOVA) followed by the Tukey-Kramer post-hoc test to determine differences among the groups. A *P *value of less than 0.05 is considered as statistically significant.

## Competing interests

The authors declare that they have no competing interests.

## Authors' contributions

YM, NE, TK, TY, HI and RO are responsible for experimental design. YM and HS are responsible for performance of behavioral test. YM, HS, TK and SU are responsible for performance of Western blot, PCR and NADPH diaphorase staining. YM, NE, HS, TK and RO are responsible for writing the manuscript. All authors read and approved the final manuscript.
